# *Mycobacterium bovis* and *M*. *tuberculosis* in Goats, Nigeria

**DOI:** 10.3201/eid1512.090319

**Published:** 2009-12

**Authors:** Simeon I. Cadmus, Hezekiah K. Adesokan, Akinbowale O. Jenkins, Dick van Soolingen

**Affiliations:** University of Ibadan, Ibadan, Nigeria (S.I. Cadmus, H. K. Adesokan); University of Pretoria, Onderstepoort, Pretoria, South Africa (A.O. Jenkins); National Institute for Public Health and the Environment, Bilthoven, the Netherlands (D. van Soolingen)

**Keywords:** Goats, tuberculosis and other mycobacteria, molecular typing, Mycobacterium bovis, Mycobacterium tuberculosis, zoonoses, bovine tuberculosis, bacteria, Nigeria, letter

**To the Editor:** Documentation of possible tuberculosis (TB) in goats in Nigeria was reported by Ojo (*1*) on the basis of gross lesions without culture confirmation. Livestock owners in Nigeria normally graze cattle and goats together, and this practice poses a high risk for transmission of bovine TB among these animals (*1*). This practice is especially a threat to goats in Nigeria because several reports have described bovine TB in cattle in Nigeria (*2**–**5*). However, reports of diagnosis of TB in goats in Nigeria are lacking.

Molecular epidemiologic techniques such as deletion typing and spoligotyping have been used to characterize members of the *Mycobacterium tuberculosis* complex (MTC) and to provide information on transmission of mycobacterial diseases between animals and humans (*6*). However, because of limited resources and lack of expertise, these techniques are not commonly used in most developing nations such as Nigeria, where TB is endemic (*3*).

Because slaughterhouses provide excellent opportunities for detecting diseases of economic and public health importance, we investigated the presence of mycobacteria in slaughtered goats with lesions suggestive of TB. The investigation was conducted at the Bodija Municipal Abattoir in Ibadan in southwestern Nigeria over a period of 6 months. Slaughtered goats were obtained from local herds and herds in northern Nigeria. A total of 10,389 male and female goats of 2 breeds (West African Dwarf and Red Sokoto) and 1–2 years of age were slaughtered; 1,387 were inspected for gross lesions of TB.

Of 1,387 animals screened, 62 (4.47%) had lesions suggestive of TB in the liver, lungs, and mesenteric lymph nodes. Five (0.36%) goats were confirmed positive by culture as described (*2*). Deletion typing (*6*) with the RD9 deletion was used to distinguish *M*. *tuberculosis* from other members of the MTC. Those isolates with a deletion in this region were further investigated with primers specific for RD4. This reaction distinguishes between *M*. *bovis*, *M*. *caprae*, and other members of the MTC. Spoligotyping was performed as described (*7*) to type an *M*. *tuberculosis* isolate from a goat after identification of this bacterium by deletion typing.

We isolated 4 strains of *M*. *bovis* and 1 strain of *M*. *tuberculosis* from the goats (Table). Spoligotyping identified the *M*. *tuberculosis* isolate as belonging to the East African Indian (EAI)–5 family in the SpolDB4 database. All *M*. *bovis* isolates were *M*. *bovis bovis*, not *M*. *bovis caprae*, according to their deletion typing profile (*6*). One *M*. *bovis* isolate was obtained from a male goat; the 3 remaining *M*. *bovis* isolates and the *M*. *tuberculosis* isolate were obtained from female goats.

**Table:** Results of deletion typing for *Mycobacterium tuberculosis* and *M*. *bovis* in goats, Nigeria*

Region of difference	*M. tuberculosis*	*M. bovis*
RD1	+	+
RD4	+	–
RD9	+	–
RD12	+	–

*+, present; –, absent.

Epidemiologic inferences can be made from the results of our study. First, *M*. *bovis*, which is naturally found in cattle, was isolated from 4 slaughtered goats. Although *M*. *bovis caprae* was the *M*. *bovis* variant most frequently isolated from goats in some areas (*8*), in our study, only *M*. *bovis bovis* was isolated. This finding is consistent with results reported by Crawshaw et al. (*9*), and suggests transmission from cattle, rather than transmission from the goat reservoir. Second, because the infected goats were adult females, infection may be transmitted to their offspring. Third, *M*. *tuberculosis* was isolated from a goat. Its presence in this goat may have been caused by direct transmission from humans because this bacterium may be a natural pathogen of humans.

Transmission caused by close cohabitation of goats and humans with advanced TB may occur, given the endemic nature of TB in humans in Nigeria (*10*). TB cases caused by EAI strains have been found in humans in southwestern Nigeria (*4*; S.I. Cadmus et al., unpub. data), a finding that supports zoonotic transmission of this organism from humans to goats. However, different lineages of *M*. *tuberculosis* may vary in host range, and EAI genotype strains may be adapted to human and animal hosts. Conversely, human-to-animal transmission of *M*. *tuberculosis* has been reported in Nigeria relative to infection in cattle (3,*4*). Thus, confirmation of TB in goats supports the possibility of risk for TB transmission between humans and animals in Nigeria.

This study should be interpreted in the context of its limitations. Because the sources of the animals were unknown, we could not determine whether the organisms were imported from a neighboring country (*3*). In addition, we lacked information on the breed and condition of the animals. However, we have identified *M*. *tuberculosis* and TB in goats in Nigeria. Additional studies of other slaughterhouses in Nigeria are needed to confirm our results.
